# Redox Properties of Structural Fe in Clay Minerals: 5. The Role of Fe in the Tetrahedral Sheets of Synthetic Smectites

**DOI:** 10.1021/acs.est.6c07316

**Published:** 2026-10-01

**Authors:** Vineeth Pothanamkandathil, Fabien Baron, Anke Neumann, Jagannath Biswakarma, Michael Sander, Thomas B. Hofstetter, Meret Aeppli

**Affiliations:** † 27218 School of Architecture, Civil and Environmental Engineering (ENAC), École Polytechnique Fédérale de Lausanne (EPFL), 1015 Lausanne, Switzerland; ‡ 27077 Université de Poitiers, CNRS, IC2MP, 86073 Poitiers, France; § 28498 PSI Center for Nuclear Engineering and Sciences, 5232 Villigen PSI, Switzerland; ∥ School of Engineering, 1980 Newcastle University, Newcastle Upon Tyne NE1 7RU, United Kingdom; ⊥ Eawag, 27219 Swiss Federal Institute of Aquatic Science and Technology, 8600 Dübendorf, Switzerland; # 28499 Institute of Biogeochemistry and Pollutant Dynamics (IBP), ETH Zürich, 8092 Zürich, Switzerland

**Keywords:** smectite, synthetic nontronite, structural iron, tetrahedral iron, redox properties, mediated electrochemical analysis, electron-transfer kinetics, standard reduction potential, biogeochemical cycling, subsurface redox processes

## Abstract

Iron-bearing smectite clay minerals are important redox-active phases in soils and sediments. Yet, the influence of iron substitution in the tetrahedral sheets (^Tet^Fe) of smectites on their redox properties remains poorly understood. Here, we investigated the repeated reduction and re-oxidation of synthetic Fe-rich smectites, known as synthetic nontronites (SyN), with constant content of Fe in the octahedral sheet (^Oct^Fe) but variable initial content of ^Tet^Fe. The redox properties of redox-cycled smectites were characterized using mediated electrochemical analysis. The resulting Fe^2+^/Fe_Total_ ratios determined over a wide range of reduction potentials were analyzed using a process-based model to extract thermodynamic and kinetic descriptors of the interfacial electron transfer and subsequent charge redistribution processes that occur during the redox reaction. The fitted standard reduction potentials and apparent diffusion coefficients for charge redistribution were insensitive to ^Tet^Fe content, indicating that thermodynamics and bulk charge transport within the smectite crystal are predominantly governed by the Fe–O–Fe networks in the octahedral sheet. Conversely, samples containing ^Tet^Fe exhibited fitted interfacial electron-transfer rate constants approximately an order of magnitude higher than the ^Tet^Fe-free sample. Comparison of these thermodynamic and kinetic descriptors between synthetic nontronites and natural reference smectites showed that parameter values converge within a narrow range across iron-rich dioctahedral smectites. These model-based results highlight the distinct roles of ^Oct^Fe and ^Tet^Fe in controlling smectite redox reactivity in subsurface environments, with ^Oct^Fe governing the thermodynamics and ^Tet^Fe primarily influencing interfacial electron-transfer kinetics.

## Graphical abstract

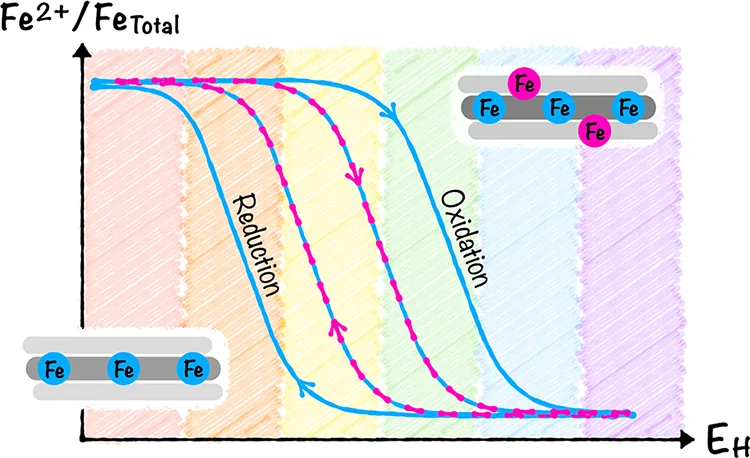

## Introduction

Smectite clay minerals containing structural iron play important roles in subsurface redox processes and thus are critical for many biogeochemical processes.^1–7^ Such smectites can serve as both electron donors and acceptors, thereby supporting anaerobic microbial respiration, contaminant transformation, as well as trace element and nutrient cycling.^3,8–17^ Unlike ferric iron oxides, Fe^3+^ reduction in smectites does not lead to reductive mineral dissolution.^16–21^ Instead, structural Fe is largely preserved within the smectite lattice as the local charge deficit caused by reduction is compensated by cation uptake and structural re-arrangement.^16–19,21^ Therefore, these clay minerals are considered a recyclable pool of redox-active Fe in subsurface environments.^22–24^

Despite structural Fe in smectites constituting a significant fraction of the total Fe pool in soils and sediments, the redox thermodynamics of Fe in this pool remain poorly described as compared to those of iron oxides, such as goethite and hematite.^16,25–27^ Assessing the redox thermodynamics in smectites is challenged by their broad compositional and structural variability. Specifically, smectites vary widely in total Fe content, the distribution of Fe between octahedral and tetrahedral sheets, the identity of neighboring cations in the octahedral sheet, and layer charge, all of which may influence their redox properties.^21^ Therefore, characterizing the redox properties of smectites and how these relate to structure is essential for understanding their role in biogeochemical processes.^10,16,17^

Characterizing redox properties is, however, not straightforward. The current conceptual view—exemplified here for Fe^3+^ reduction—implies two coupled processes operating over different time scales. While interfacial electron transfer through edge or basal sites^28^ is relatively fast, charge redistribution and cation migration in the smectite’s octahedral sheet are slow. The ensuing charge and chemical potential gradient between the surface and the bulk of the smectite particle is maintained for minutes to hours, over which particles do not attain thermodynamic equilibrium with their surrounding chemical environment.^17^ These phenomena have important implications for how smectite redox properties are measured and interpreted using mediated electrochemical analysis (MEA).^29^ This approach involves electrochemical reduction and oxidation of smectites whose Fe^2+^/Fe^3+^ ratio and thus redox state were left as-is or were altered through chemical reduction and subsequent oxidation, with dithionite and hydrogen peroxide, respectively.^23,30–32^ These studies with widely-used natural reference smectites, namely, SWy-2, SWa-1, NAu-1, and NAu-2, revealed that all structural Fe was redox-active across a wide range of standard reduction potentials, *E*_H_, between −0.6 to +0.4 V vs SHE.^23,31,32^ The relatively short duration of MEA, typically 30–60 min, and the inability of smectite particles to fully equilibrate with the applied potential prompted the introduction of a more comprehensive model that accounted for both thermodynamic and kinetic aspects associated with Fe redox reactions of smectites.^29^ This model now accounts for both the electron-transfer kinetics and structural re-arrangement as well as the thermodynamic driving force and reconciles divergent observations made with redox-manipulated smectites.

While the work of Pothanamkandathil et al.^29^ has advanced our understanding of smectite redox behavior, it primarily focused on the contributions from Fe in the octahedral sheet of smectites (i.e., octahedrally coordinated Fe, ^Oct^Fe). In fact, ^Oct^Fe typically accounts for nearly all of the available electron-exchange capacity in the studied smectites.^23^ The potential role of Fe in the tetrahedral sheets (i.e., tetrahedrally coordinated Fe, ^Tet^Fe) in determining the redox behavior of smectites, however, remains unknown. This disparity largely reflects the limited occurrence of ^Tet^Fe in reference smectites, where it is confined to a small subset of minerals (e.g., NAu-2) and typically constitutes a minor fraction of the total structural Fe. Moreover, the absence of reference materials with known ^Tet^Fe content complicates efforts to study its contribution to the overall redox activity of a smectite.^33–36^ Despite its low abundance, even small amounts of tetrahedral substitution of Si^4+^ by Fe^3+^ could influence electron transfer between aqueous reductants and oxidants and smectite particles. Critical factors include an increase of surface contact points for electron exchange, the provision of additional electron conduction pathways within the smectite lattice, and the incompatibility of structural ferrous iron, Fe_s_^2+^, with coordination in the tetrahedral sheet.^28,33,34,37^

In this study, we make use of the so far unexplored opportunity to disentangle the effect of tetrahedrally substituted Fe on smectite reactivity by evaluating the redox reactions of synthetic smectites rich in ^Tet^Fe.^38,39^ Unlike the high variability in Fe content and coordination environment observed in reference smectite minerals, synthetic specimens enable precise control over these parameters, thus facilitating a direct assessment of the role of ^Tet^Fe in smectite redox reactions.^38–41^ Here, we posit that the presence of Fe in the tetrahedral sheets alters the redox properties of smectites by (i) shifting the thermodynamic stability of the mineral lattice and thereby modifying the standard reduction potential (*E*_H_^0^) and (ii) potentially enhancing electron-transfer kinetics by improving the accessibility of structural Fe to external reductants and oxidants.

To evaluate these hypotheses, we investigated a series of synthetic nontronites (SyN) that were structurally characterized in a companion paper.^42^ In this series of synthetic nontronites, the abundance of ^Oct^Fe was held constant at 2 atoms per half-unit cell while the ^Tet^Fe content was systematically varied between 0 and 1.09 atoms per half-unit cell, providing an ideal sample set for studying the influence of ^Tet^Fe on smectite redox behavior. Note that such idealized compositions lack the structural heterogeneity, mineralogical impurities, variable layer charge, and diverse interlayer cation compositions that characterize natural smectites.^1,3,36^ The trends reported here therefore describe the effect of ^Tet^Fe within a compositionally controlled series, and their extension to natural specimens requires that these additional sources of variability be taken into account. We investigated these nontronites using MEA to generate Fe_s_^2+^/Fe_Total_–*E*_H_ relationships following established procedures for redox-cycled smectites.^32^ Subsequently, the measured Fe_s_^2+^/Fe_Total_–*E*_H_ relationships were analyzed using the model introduced by Pothanamkandathil et al.^29^ to extract relevant redox properties such as standard reduction potentials, heterogeneous rate constants for electron transfer and structural re-arrangements, and apparent diffusion coefficients. Finally, these parameters were then used to compare redox properties across the synthetic nontronites investigated in this study and with previously studied reference smectites.

## Materials and Methods

### Solutions

All solutions and mineral suspensions were prepared with doubly deionized water (DDW, resistivity >18.2 MΩ·cm, Barnstead Nanopure Diamond Water Purification System) and purged with ultrahigh purity N_2_ for at least 3 h prior to transfer into an anoxic glovebox (N_2_ atmosphere, <2 ppm of O_2_; Unilab 2000, MBraun). Solutions for MEA measurements contained 0.01 M NaCl as electrolyte and 0.01 M organic pH buffer (3-morpholinopropane-1-sulfonic acid (MOPS; p*K*_a_ = 7.2)).

### Clay Minerals

Five synthetic nontronites, hereafter referred to as SyN-1, SyN-2, SyN-3, SyN-4, and SyN-5, were synthesized and characterized as described in the companion study by Baron et al.^42^ The structural composition and purity of the nontronites were evaluated using X-ray diffraction (XRD), Mössbauer spectroscopy, infrared (IR) spectroscopy, inductively coupled plasma optical emission spectroscopy (ICP-OES), and transmission electron microscopy (TEM).^42^ Based on these analyses, the total Fe content in these samples ranged from 25 to 37 wt % (Table S1). SyN-1 contained Fe exclusively in the octahedral sheet (1.91 atoms per half-unit cell), with trace amounts of Al^3+^ being present in both tetrahedral (0.38 atoms per half-unit cell) and octahedral sheets (0.09 atoms per half-unit cell) (Table S1). This Al^3+^ substitution was required to stabilize the tetrahedral sheet in the absence of sufficient amounts of tetrahedral Fe and to maintain the structural integrity of the 2:1 layer.^42^ In contrast, SyN-2, -3, -4, and -5 all contained two atoms of Fe per half-unit cell in the octahedral sheet with varying amounts of Fe (i.e., 0.38, 0.60, 1.04, and 1.09 atoms per half-unit cell) in the tetrahedral sheets (Table S1). The structural formula and the ^Tet^Fe and ^Oct^Fe distribution for each sample are provided in Table S1. TEM images reported by Baron et al.^42^ showed particles of approximately 100–200 nm forming foliated aggregates, with interlayer spacings of about 1 nm and no evidence of amorphous or secondary crystalline phases.

The SyN samples were subjected to controlled redox manipulations following the procedure described in Baron et al.^42^ Briefly, as-synthesized SyN suspensions were chemically reduced with sodium dithionite under anoxic conditions to generate “reduced” samples, and aliquots of these reduced suspensions were subsequently re-oxidized with hydrogen peroxide to obtain “re-oxidized” samples.^23,31^ A subset of the re-oxidized samples was reduced again with dithionite, yielding “re-reduced” samples. A schematic of the experimental protocol employed on the SyN samples is depicted in Figure S1. These treatments enabled comparison of as-synthesized, reduced, re-oxidized, and re-reduced SyN samples, and allowed us to assess how redox cycling altered the structure and redox behavior of ^Tet^Fe and ^Oct^Fe. Note that the re-oxidized SyN-2 sample was lost during transport and could not be electrochemically analyzed. Consequently, complete extraction of redox parameters for SyN-2 using the process-based model was not possible, as this requires both reduction and oxidation curves obtained from the reduced, re-oxidized, and re-reduced samples (see Model Description and Data Analysis section below). However, SyN-2 was included in our analysis of the as-synthesized SyN samples.

### Mediated Electrochemical Analyses

Mediated electrochemical reduction (MER) and oxidation (MEO) experiments were conducted to quantify the number of electrons transferred to or from structural iron in synthetic nontronites at constant applied potentials ranging from −0.57 V to +0.70 V vs SHE, in pH 7.50-buffered solutions (pH 6.50 for analyses with the electron transfer mediator ABTS [2,2′-azino-bis­(3-ethylbenzothiazoline-6-sulfonic acid)]) following established protocols.^23,30,32^ We report electrochemical data as ratios of Fe_s_^2+^/Fe_Total_, which were determined by normalizing *n*_Fe_MEO_^II^_ or Fe_Total_ – *n*_Fe_MER_^III^_ by Fe_Total_. Fe_Total_ is the number of Fe atoms in the sample added to the electrochemical cells, which was calculated as the sum of the electron-accepting capacity (measured at an applied *E*_H_ = −0.57 V) and electron-donating capacity (measured at an applied *E*_H_ = 0.70 V) of the sample (see Section S2 for details). Fe_Total_ values determined electrochemically were in good agreement with Fe contents measured independently by SEM-EDS.^42^

### Model Description and Data Analysis

#### Model Implementation

Nonequilibrium Fe_s_^2+^/Fe_Total_–*E*_H_ relationships obtained using MER and MEO were simulated using a model introduced by Pothanamkandathil et al.^29^ and subsequently applied by Liu et al.^43^ that explicitly took into account (i) the thermodynamic driving force for the redox reaction, (ii) the interfacial electron-transfer kinetics, and (iii) the redistribution of charges within the smectite lattice. For the sake of brevity, we only present the governing equations that directly relate to the redox properties extracted from the experimental redox curves here.

The redox reaction involving the structural iron in the smectite can be defined using the following simplified half-reaction

1 $${\mathrm{Fe}}_{s}^{3+}+e^{-}+{\mathrm{Cat}}_{\mathrm{aq}}^{+}⇌{\mathrm{Fe}}_{s}^{2+}{\mathrm{Cat}}_{s}^{+}$$

where Fe_s_^3+^ and Fe_s_^2+^ represent the structural ferric and ferrous ions in the smectite crystal, and Cat^+^ represents a monovalent cation that can reversibly intercalate into the smectite interlayers. The thermodynamic driving force that dictates whether eq 1 proceeds in the forward or backward direction is determined by the equilibrium state of the smectite–water interface and is defined by the Nernst–Frumkin equation

2 $$E_{H,\mathrm{eq}}=E_{H}^{0}-\frac{\mathrm{RT}}{\mathrm{nF}}\ln\left(\frac{\theta }{1-\theta }\right)+\frac{\mathrm{RT}}{\mathrm{nF}}\ln\left\{{\mathrm{Cat}}_{\mathrm{aq}}^{+}\right\}+\frac{\mathrm{RT}}{F}g\left(0.5-\theta \right)$$

where *E*_H_^0^ (V vs SHE) is the standard reduction potential (at Fe_s_^2+^/Fe_Total_ = 0.5) of the smectite at pH 7.5, *R* is the ideal gas constant (8.314 J·K^–1^·mol^–1^), *T* (K) is the temperature, *n* is the number of electrons involved in the redox half-reaction, and *F* is Faraday’s constant (96,485 C·mol^–1^). The Fe_s_^2+^/Fe_Total_ ratio at the smectite–water interface is represented by *θ*, and *g* is a dimensionless parameter that accounts for the broadening of the Fe_s_^2+^/Fe_Total_–*E*_H_ relationship (redox curve) due to intrinsic heterogeneity in the coordination environments of the Fe sites.

The electron flux (*r*_SA_, mol·cm^–2^·s^–1^) of electron transfer between the smectite particle and the mediator at the interface in response to an applied potential *E*_H_ (V vs SHE) is described by the following set of equations^29^

3 $$r_{\mathrm{SA}}=\Gamma_{\mathrm{Total}}\cdot \left(k_{f}\cdot \left[{\mathrm{Cat}}_{\mathrm{aq}}^{+}\right]\cdot \left(1-\theta \right)-k_{b}\cdot \theta \right)$$

4 $$k_{f}=\frac{k_{0}}{{\left[{\mathrm{Cat}}_{\mathrm{aq}}^{+}\right]}_{\mathrm{ref}}}\cdot \exp\left(-\alpha \cdot \frac{\mathrm{nF}}{\mathrm{RT}}\cdot \left(E_{H}-E_{H,\theta }\right)\right)$$

5 $$k_{b}=k_{0}\cdot \exp\left(\left(1-\alpha \right)\cdot \frac{\mathrm{nF}}{\mathrm{RT}}\cdot \left(E_{H}-E_{H,\theta }\right)\right)$$

where Γ_Total_ (mol·cm^–3^) is a scaling factor that represents the number of Fe surface sites available in a unit volume of clay suspension. The potential-dependent forward (*k*_f_) and backward (*k*_b_) rate constants are derived from the standard heterogeneous rate constant (*k*_0_, cm·s^–1^) using eqs 4 and 5 and scale exponentially with the potential difference (*E*_H_ – *E*_H,θ_). The reference cation activity ([Cat_aq_^+^]_ref_ = 1 × 10^–3^ mol·cm^–3^) is employed to maintain dimensionality between both the forward and backward reactions in eq 3. In eqs 4 and 5, the transfer coefficient (0 ≤ α ≤ 1) describes the structural similarity of the reaction intermediate to either the reactant or the product in eq 1, and *E*_H,θ_ represents the reduction potential at the surface of the smectite particle, which varies as a function of surface coverage

6 $$E_{H,\theta }=E_{H}^{0}+\frac{\mathrm{RT}}{F}g\left(0.5-\theta \right)$$

Together, *k*_0_ and α describe the interfacial electron transfer while taking the surface composition of smectite into account through *E*_H,θ_ and *g*. To link the surface regeneration of redox-active Fe sites at the interface with the slow redistribution of charged species within the smectite lattice, we employed a finite element one-dimensional diffusion model based on Fick’s laws

7 $$J=-D\cdot \Gamma_{\mathrm{Total}}\cdot \frac{d\theta }{dx}$$

8 $$\frac{d\theta }{dt}=-D\cdot \frac{d^{2}\theta }{dx^{2}}$$

where *J* (mol·cm^–2^·s^–1^) is the flux of charge across the smectite–water interface and is defined by the boundary condition *J* = *r*_SA_. The redistribution of charges, which can occur either via cation migration or electron hopping through Fe–O–Fe linkages, is captured by the apparent diffusion coefficient *D* (cm^2^·s^–1^). The temporal and spatial resolutions of the finite element model are represented by d*t* (s) and d*x* (cm), respectively.

#### Simulation and Fitting of Redox Curves

Depending on whether the smectite samples were analyzed in their as-synthesized state or chemically pre-treated (with dithionite and/or hydrogen peroxide) prior to electrochemical analysis, two different modeling strategies were used to interpret the Fe_s_^2+^/Fe_Total_–*E*_H_ curves.

##### Analysis of As-Synthesized SyN Samples

The reduction curves obtained from MER experiments on as-synthesized SyN samples were fitted to eq 2 without incorporating the kinetics of interfacial electron transfer or internal charge redistribution. Therefore, an apparent reduction potential (*E*_H_^Ø^) was determined for the as-synthesized SyN samples, which is an empirical descriptor of the mid-point (i.e., Fe_s_^2+^/Fe_Total_ = 0.50) of the measured reduction curve rather than a thermodynamic descriptor of electron transfer to/from structural Fe; the latter is captured by *E*_H_^0^ (see next subsection). This simplified approach was necessitated by practical limitations in post-MER sample recovery. Due to the low volume of smectite suspension spiked into the electrochemical cell during MER, it was not possible to recover sufficient solid material to conduct subsequent MEO experiments. Because oxidation curves could not be generated for the as-synthesized SyN samples, the parameters for interfacial electron transfer and internal charge redistribution kinetics could not be extracted. The parameters *E*_H_^Ø^ and *g* were determined by minimizing the sum of least-squared differences between the experimental and simulated Fe_s_^2+^/Fe_Total_–*E*_H_ relationships using the L-BFGS-B optimization algorithm.^44^ During this process, the data were weighted by the inverse of the slope squared, (d*E*_H_/dθ)^−2^, to reduce the bias of regions where a small change in θ produced a large change in *E*_H_ (i.e., θ ≥ 0.95 or θ ≤ 0.05).

##### Analysis of Chemically Treated SyN Samples

For samples that were subjected to chemical treatment prior to MEA (i.e., SyN samples reduced with dithionite and samples that were initially reduced using dithionite and subsequently re-oxidized using hydrogen peroxide), the set of measured reduction and oxidation curves was fitted to the model to extract the redox parameters for each SyN sample. Specifically, the parameters *E*_H_^0^, *k*_0_, *D*, α, and *g* were extracted for each SyN sample by simultaneously fitting the experimental reduction and oxidation curves that were obtained from MER experiments on re-oxidized and MEO experiments on reduced/re-reduced samples, respectively. Note that the experimental oxidation curves for both the reduced and re-reduced SyN samples were collectively fitted using a single simulated oxidation curve for each SyN sample. This was done due to the high degree of overlap between the oxidation curves of the reduced and re-reduced samples. Additionally, accurate simulations of the redox curves required the definition of an initial Fe_s_^2+^/Fe_Total_ ratio in the redox-manipulated samples prior to the electrochemical measurements, which we determined electrochemically (see above). A description of how the initial Fe_s_^2+^/Fe_Total_ ratio was calculated is provided in Section S3 of the supporting information. The fitting was implemented using a two-step optimization approach that minimized the sum of least-squared differences between the experimental and simulated Fe_s_^2+^/Fe_Total_–*E*_H_ relationships. The two-step optimization process involved an initial global search using a genetic algorithm to identify the most likely parameter values while avoiding local minima.^45^ This was followed by a local refinement using the L-BFGS-B algorithm to ensure a precise fit.^44^ To assess the uncertainty associated with the fitted parameters, a bootstrap resampling method was used to add random noise to the data and calculate 95% confidence intervals for each fitted parameter.^46^ Briefly, Gaussian noise with a standard deviation equal to that of the best-fit residuals was added to the measured data, and the fitting was repeated over 100 iterations to determine the spread in the resulting parameter values. Additionally, the identifiability of the fitted parameters was assessed by varying each parameter systematically around its optimal value while holding the remaining parameters at their best-fit values (Figure S2).

##### Principal Component Analysis

Principal component analysis (PCA) was used to evaluate the relationships between the fitted redox parameters and the residual ^Tet^Fe content of the redox-manipulated SyN samples. The fitted redox parameters included the standard reduction potential (*E*_H_^0^), the interaction parameter (*g*), and the heterogeneous rate constant (*k*_0_), with *k*_0_ represented as log *k*_0_. To account for the uncertainty in our fitted *E*_H_^0^, *g*, and log *k*_0_ values, we performed a Monte Carlo simulation (*N* = 10,000) to generate a distribution of fitted parameters for each sample as the input for the PCA. All simulations and fitting procedures were implemented in the R statistical computing environment,^47^ as documented earlier.^29^

## Results and Discussion

### Redox Characterization of As-Synthesized Synthetic Nontronites

The as-synthesized SyN samples varied systematically in tetrahedral Fe (^Tet^Fe) content from 0 to 1.09 atoms per half-unit cell, allowing us to assess the effect of ^Tet^Fe on smectite redox properties (Figure 1A).^42^ The redox properties of the as-synthesized SyN samples obtained through mediated electrochemical reduction (MER) experiments are shown in Figure 1B over applied potentials between −0.6 and +0.6 V vs SHE. All five SyN samples exhibited trends similar to the reduction curves previously observed for natural Fe-containing smectites, with the extent of reduction (i.e., the Fe_s_^2+^/Fe_Total_ ratio) increasing with decreasing applied *E*_H_.^32^ Moreover, complete reduction of structural Fe_s_^3+^ to Fe_s_^2+^ occurred at −0.6 V vs SHE for all five SyN samples, indicating that both tetrahedral and octahedral Fe were redox-active and reduced by the mediator at low *E*_H_ (Figure 1B). All five SyN samples displayed similar responses in reduction extents with *E*_H_ between −0.2 and −0.5 V vs SHE, despite having different ^Tet^Fe contents, suggesting that variations in Fe content or site distribution had little impact on the *E*_H_ at which the Fe_s_^3+^ was reduced.

**Figure 1:**
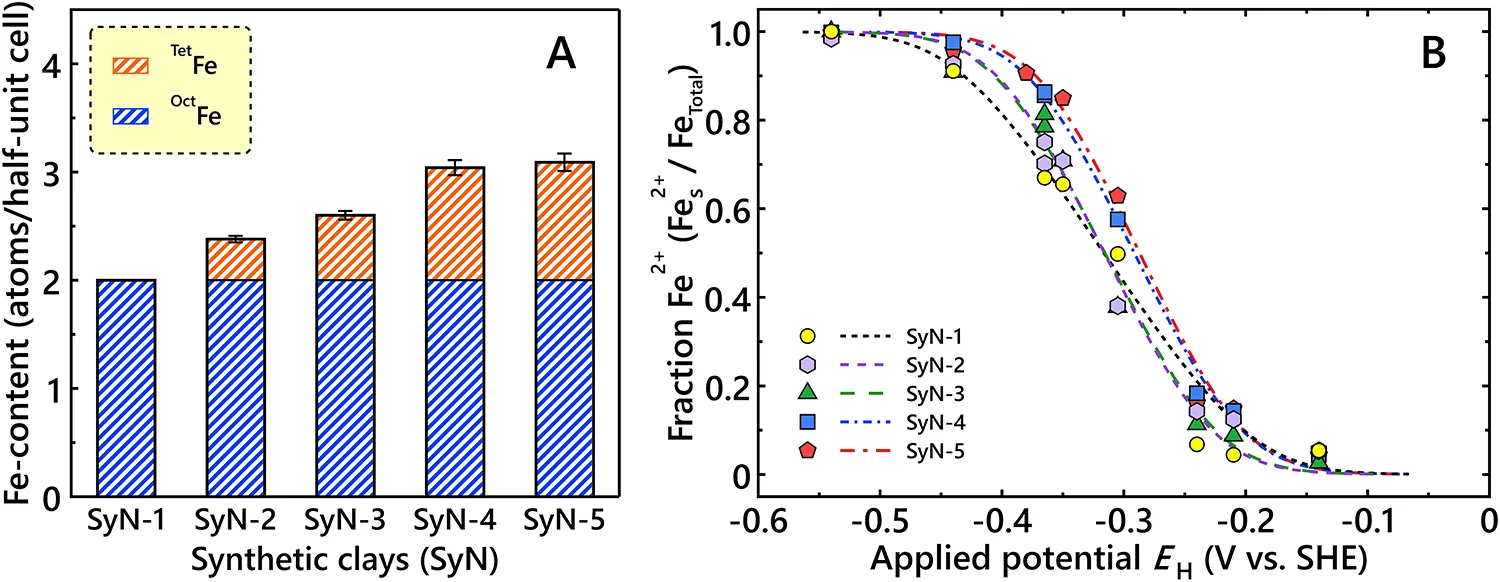
(A) Tetrahedral Fe content per half-unit cell (atoms) for the as-synthesized synthetic nontronites (SyN), as quantified using X-ray diffraction measurements. (B) Redox curves of as-synthesized synthetic nontronites obtained using MER.

To determine the redox properties of the SyN samples, we fitted the reduction curves in Figure 1B to eq 2. We note that only reduction curves were available as the as-synthesized SyN samples contained only Fe_s_^3+^. Fitted parameters are hence restricted to the apparent reduction potential (*E*_H_^Ø^) and apparent interaction parameter (*g* ^Ø^), summarized in Table 1. No kinetic or transport parameters could be determined, as this would have required both reduction and oxidation curves.^29^

**Table 1:** Summary of Fitted Apparent Thermodynamic (*E*_H_^Ø^ and *g* ^Ø^) Parameters for SyN Samples, Obtained by Fitting Reduction Curves of As-Synthesized SyN Samples to Equation 2

Smectite	^Tet^Fe (atoms per half-unit cell)	*E*_H_^Ø^ (V vs SHE)	σ (*E*_H_^Ø^) (V vs SHE)	*g* ^Ø^	*g* ^Ø^-range	*r* ^2^
SyN-1	0.00	–0.198	0.095	5.69	5.32 to 9.69	0.89
SyN-2	0.38	–0.197	0.075	4.75	0.75 to 6.16	0.97
SyN-3	0.60	–0.197	0.073	3.35	3.15 to 7.35	0.88
SyN-4	1.04	–0.173	0.078	3.11	–0.89 to 4.20	0.99
SyN-5	1.09	–0.169	0.078	2.81	–1.19 to 3.74	0.98

The *E*_H_^Ø^ values increase from −0.198 V for SyN-1 to −0.169 V vs SHE for SyN-5. While this shift occurs in the direction of increasing ^Tet^Fe, the range of fitted *E*_H_^Ø^ values (29 mV, Table 1) is narrow, particularly when compared to the standard deviations associated with the fitted values (∼80 mV, Table 1). Furthermore, because these parameters were derived solely from reduction curves, it remains unclear whether this subtle shift reflects a true thermodynamic change in Fe reduction potential or a kinetic effect related to the accessibility of tetrahedral Fe during MER. By contrast, the apparent interaction parameter, *g* ^Ø^, exhibited an inverse trend; the value for SyN-1 was higher (*g* ^Ø^ = 5.69) than those of SyN-2, -3, -4, and -5 (ranging from 2.81 to 4.75, Table 1), indicating that SyN-1 spans a wider redox-active range than the other four samples (Figure 1B). This trend suggests that the presence of ^Tet^Fe has a narrowing effect on the redox-active range of the SyN samples. Additionally, the presence of Al^3+^ in both tetrahedral and octahedral sites of SyN-1 (Table S1) can alter the local Fe coordination environment and broaden the *E*_H_ distribution of the Fe_s_^2+/3+^ redox couple. However, this trend should be interpreted with caution, due to the *g* ^Ø^-range overlap between samples (Table 1).

### Effect of Redox Cycling on the Redox Properties of Synthetic Nontronites

The experimental reduction and oxidation curves for the redox-manipulated SyN samples are depicted in Figure 2. For all four SyN samples, the reduction curves of the re-oxidized samples were visibly distinct from those of the as-synthesized samples (red vs yellow markers in Figure 2). Specifically, the re-oxidized samples exhibited reduction curves that shifted toward more positive reduction potentials and spanned a wider potential range compared to their as-synthesized counterparts (Figure 2). This behavior is consistent with previous observations for iron-rich reference smectites, in which dithionite reduction followed by re-oxidation altered the Fe_s_^2+^/Fe_Total_–*E*_H_ relationships.^31,32^ Additionally, the oxidation curves of both reduced and re-reduced samples shifted to significantly more positive potentials than the reduction curves (blue vs yellow and red markers in Figure 2), indicating pronounced redox hysteresis, as previously demonstrated for reference smectites.^31,32^ However, unlike the reduction curves of the as-synthesized and re-oxidized samples, the oxidation curves of the (re-)­reduced samples displayed a high degree of overlap (light and dark blue markers in Figure 2).

**Figure 2:**
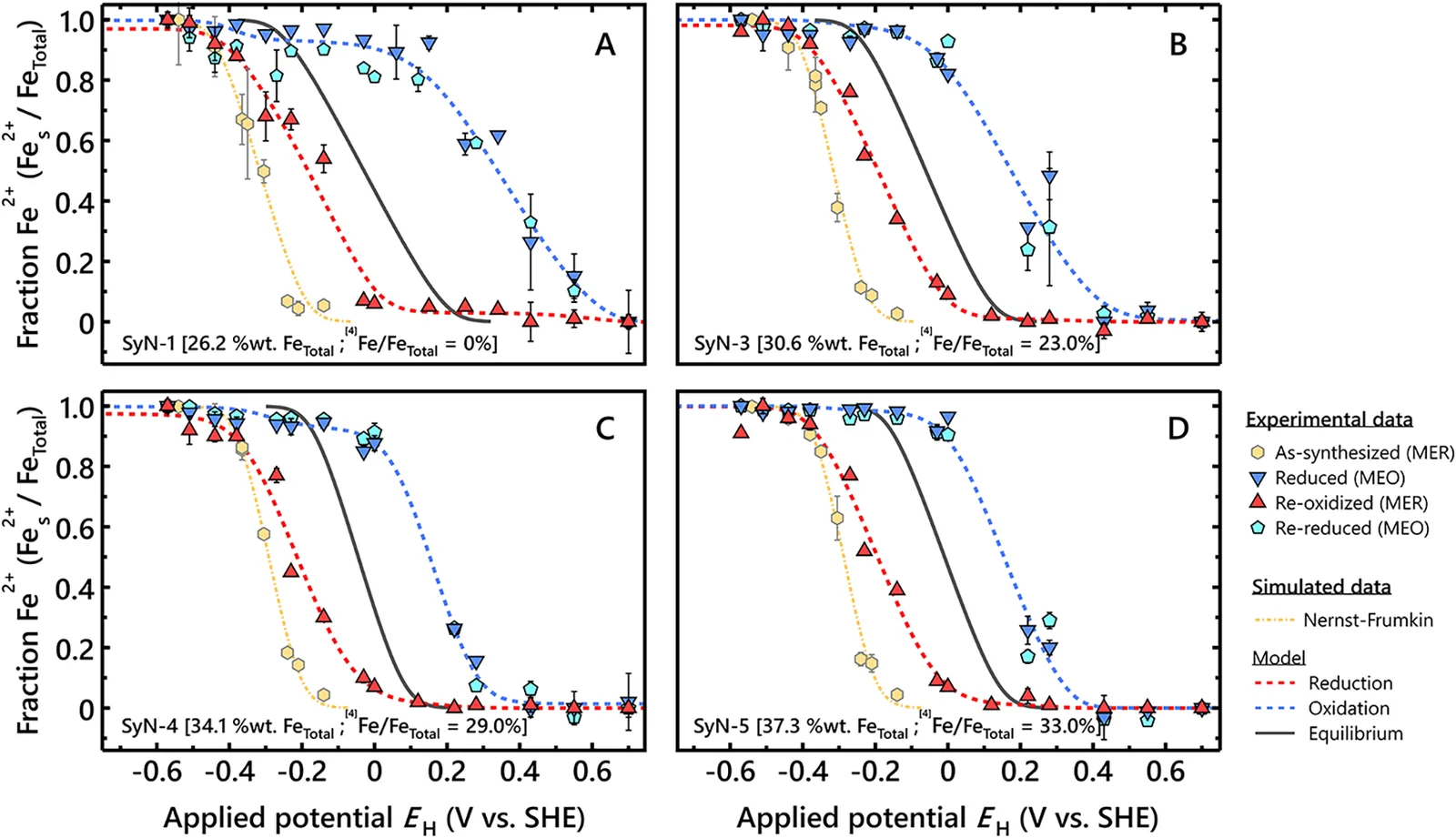
Experimental and fitted redox curves for (A) SyN-1, (B) SyN-3, (C) SyN-4, and (D) SyN-5. The red and blue markers represent experimentally determined reduction and oxidation curves obtained by conducting MER and MEO on redox-manipulated samples, respectively. The corresponding dashed lines indicate the model fits for each curve. The solid black line represents the extracted equilibrium Fe_s_^2+^/Fe_Total_–*E*_H_ relationship. For comparison, yellow markers denote reduction curves obtained from MER experiments conducted on as-synthesized SyN samples, with the respective yellow dashed line denoting the corresponding Nernst–Frumkin fit (eq 2).

Structural characterization of the reduced samples using XRD, Mössbauer spectroscopy, and IR spectroscopy in our companion paper^42^ revealed that a significant portion of the ^Tet^Fe was lost from the smectite lattice following the initial dithionite reduction, resulting in a residual ^Tet^Fe content that decreased from 0.60–1.09 (SyN-3, -4, and -5 in Table S1) to 0.03–0.27 atoms per half-unit cell (Figure 3A). Further characterization of the re-oxidized and re-reduced samples showed that the residual ^Tet^Fe content remained largely unchanged (Figure 3A), indicating that the SyN samples had attained a stable structural configuration following the first dithionite reduction step.^42^ These findings from our companion paper provide a structural basis for the trends observed in Figure 2. Specifically, the ^Tet^Fe loss and associated compositional and structural changes during the first dithionite reduction account for the divergence between the reduction curves of the as-synthesized and re-oxidized samples (red vs yellow markers in Figure 2). In contrast, the convergence of the oxidation curves of the (re-)­reduced samples reflects the structural stabilization of the smectite following the initial reduction (light vs dark blue markers in Figure 2).

**Figure 3:**
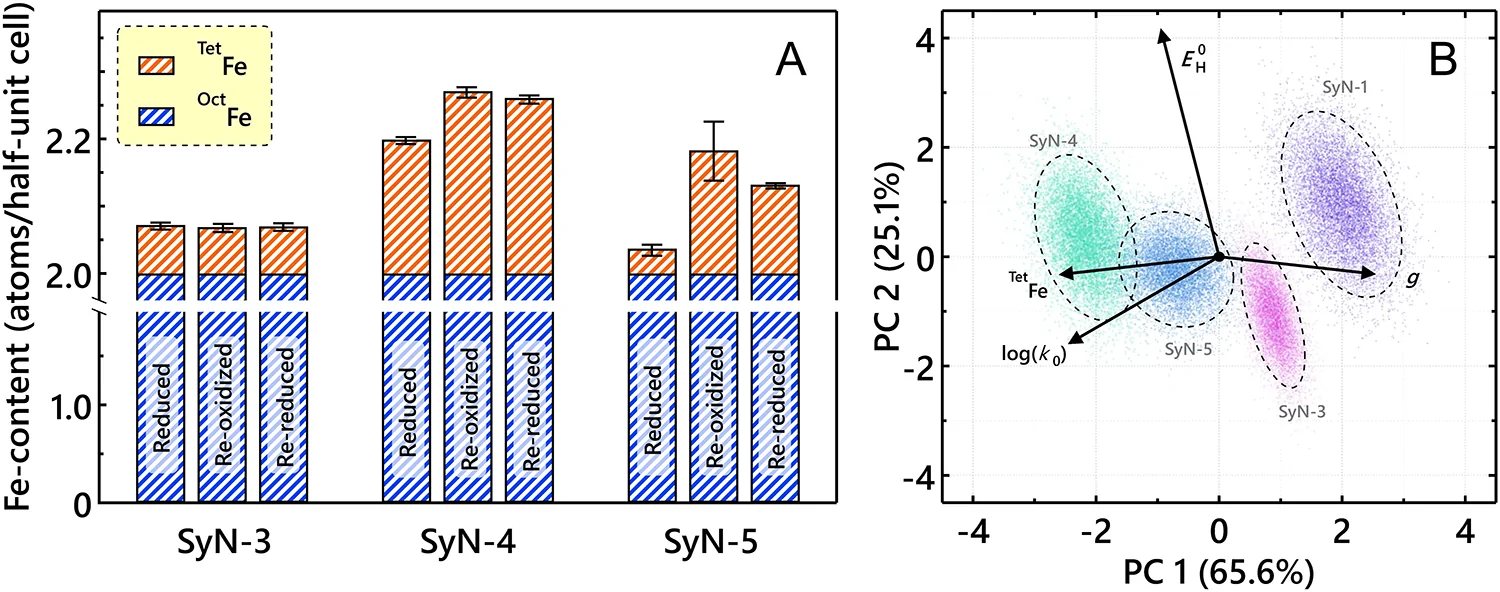
(A) Residual ^Tet^Fe and ^Oct^Fe content in redox-cycled SyN samples determined based on Mössbauer spectroscopy analysis. Note the *y*-axis split in panel A to illustrate the small differences in ^Tet^Fe per SyN specimen of ≲0.1 atoms/half-unit-cell. Error bars represent associated uncertainty in the measured ^Tet^Fe from Mössbauer spectroscopy. Data for reduced and re-reduced samples included corrections for Fe-sulfide phases (see Baron et al.^42^ for details). (B) Principal component analysis (PCA) biplot illustrating the relationships between fitted thermodynamic (*E*_H_^0^, *g*), kinetic (log *k*_0_), and structural (residual ^Tet^Fe) parameters. The alignment of the log *k*_0_ and residual ^Tet^Fe vectors is consistent with the higher rate constants determined for the ^Tet^Fe-bearing samples. The data in panel B represent the projection of Monte Carlo-simulated parameter distributions (*N* = 10,000) in the PCA biplot space and reflect the uncertainty in the fitted redox parameters for each SyN sample (Table 2); the ellipses represent 95% confidence regions.

### Quantitative Analysis of Redox-Manipulated SyN Samples and Extraction of Redox Parameters

To quantify the effect of the residual ^Tet^Fe, i.e., ^Tet^Fe remaining in the clay structure after redox manipulation, on the redox behavior of the SyN samples, we fitted the experimental reduction and oxidation curves to eqs 2 to 8. This approach allowed us to extract key parameters by decoupling the thermodynamic properties—the standard reduction potential (*E*_H_^0^, V vs SHE) and interaction parameter (*g*)—from the kinetic and charge redistribution parameters, namely, the heterogeneous rate constant (*k*_0_), transfer coefficient (α), and apparent diffusion coefficient (*D*).

The simulated fits for the reduction and oxidation curves are represented in Figure 2 by the red and blue dashed lines, respectively. For all redox-manipulated samples, the model adequately captured the experimental trends, yielding high correlation coefficients (*r* ^2^ ≥ 0.98, Table 2). Furthermore, we were able to predict the Fe_s_^2+^/Fe_Total_–*E*_H_ relationship for each sample under equilibrium conditions (solid black line, Figure 2) and determine the *E*_H_^0^ values at pH 7.5 using eq 2. The *E*_H_^0^ of the redox-manipulated SyN samples ranged between 0.054 (SyN-3, Table 2) and 0.171 V vs SHE (SyN-1, Table 2). While there was no clear trend between the fitted *E*_H_^0^ values and the corresponding residual ^Tet^Fe when all four SyN samples were considered, a distinct trend emerged among the ^Tet^Fe-bearing samples (i.e., SyN-3, -4, and -5). Specifically, for SyN-3, -4, and -5, the *E*_H_^0^ value shifted to more positive potentials as the amount of residual ^Tet^Fe increased (Table 2). Additionally, the *g*-values decreased with increasing ^Tet^Fe (Table 2). In general, although the presence of residual ^Tet^Fe in the SyN samples displayed these systematic trends, its overall effect on the thermodynamics of the redox reaction was negligible. This suggests that while ^Tet^Fe may subtly modulate the local electronic environment or site-to-site interactions (reflected by the shifts in *E*_H_^0^ and *g*, Table 2), the thermodynamics of electron transfer seem to be predominantly governed by the ^Oct^Fe network.

**Table 2:** Summary of Fitted Thermodynamic (*E*_H_^0^, *g*) and Kinetic (log *k*_0_, log *D*, α) Parameters for SyN Samples, Obtained by Fitting Experimental Data to the Model with Goodness-of-Fit Denoted by *r* ^2^.^a^

	^b^Residual ^Tet^Fe	Initial Fe_*s*_^2+^/Fe_Total_ (%)		*E*_H_^0^ at pH 7.5					
Smectite	(atoms per half-unit cell)	Reduction curve	Oxidation curve	(V vs SHE)	log(*k*_0_/cm·s^–1^)	^c^log(*D*/cm^2^·s^–1^)	α	*g*	^d^ *r* ^2^
SyN-1	0.00	2	93	0.171 ± 0.046	–10.09 ± 0.10	–11.61 ± 0.05	0.57 ± 0.08	12.12 ± 1.97	0.98
SyN-3	0.07 ± 0.01	1	98	0.054 ± 0.020	–9.49 ± 0.05	–11.47 ± 0.02	0.65 ± 0.04	11.69 ± 0.88	0.98
SyN-4	0.24 ± 0.03	1	97	0.110 ± 0.022	–9.12 ± 0.12	–11.63 ± 0.04	0.47 ± 0.05	6.23 ± 1.18	0.99
SyN-5	0.12 ± 0.06	1	99	0.086 ± 0.016	–9.28 ± 0.07	–11.67 ± 0.05	0.56 ± 0.04	8.05 ± 0.74	0.99

a The initial Fe_*s*_^2+^/Fe_Total_ ratio was determined electrochemically (Section S3); values for the oxidation curves represent averages of the reduced and re-reduced samples.

b Residual ^Tet^Fe contents represent the average of the values determined by Mössbauer spectroscopy for the reduced, re-oxidized, and re-reduced samples (Figure 3A).

c Fitted log *D* values represent lower bounds, as the simulated redox curves become relatively insensitive to *D* above log *D* ≈ −11.8.

d Residual plots and root mean square errors for each fit are provided in Figure S3 and Table S3.

In contrast to the fitted thermodynamic parameters, the heterogeneous rate constants (*k*_0_) differed more markedly between SyN-1 and the ^Tet^Fe-bearing samples, with *k*_0_ values for SyN-3, -4, and -5 ranging from 3.23 × 10^–10^ to 7.58 × 10^–10^ cm·s^–1^, approximately an order of magnitude greater than that of SyN-1 (8.13 × 10^–11^ cm·s^–1^; Table 2). This increased kinetic rate is also evidenced by the lesser extent of redox hysteresis observed in SyN-3, -4, and -5 compared to SyN-1 (Figure 2). The observed increase in fitted *k*_0_ suggests that even trace amounts of ^Tet^Fe within the nontronite structure enhance the interfacial electron-transfer kinetics. Based on the hypothesized model, we speculate that ^Tet^Fe sites facilitate faster kinetics by acting as a conductive bridge between external reductants/oxidants and the underlying ^Oct^Fe network. We note, however, that SyN-1 also differs from SyN-3, -4, and -5 in containing tetrahedral Al^3+^ and trace amounts of octahedral Al^3+^ (Table S1), and with our current sample set, we cannot disregard a potential role of this substitution in lowering *k*_0_. Differences in surface charge, aggregation, or structural defects introduced by ^Tet^Fe loss could also have an influence, although highly similar crystal chemistry was reported for all redox-cycled SyN samples.^42^ In addition, part of the Fe^2+^ released from tetrahedral sites during the dithionite reduction remained associated with the solid phase (e.g., adsorbed to the surface or intercalated into the interlayers)^42^ and could have also contributed to the higher *k*_0_ values. Finally, the apparent diffusion coefficient, *D*, was similar for all samples and ranged between 2.14 × 10^–12^ and 3.39 × 10^–12^ cm^2^·s^–1^ (Table 2). Sensitivity analysis shows that the simulated redox curves degrade rapidly in goodness-of-fit for log­(*D*) < −11.8 but change little above this value (Figure S2E). Above this threshold, charge redistribution occurs faster than the interfacial electron transfer and therefore no longer acts as a rate-limiting process. These values are consequently constrained as lower bounds, since increases in *D* beyond the threshold are not discernible in the experimental data obtained using our current approach. Our model-based findings indicate that the charge redistribution process is relatively fast compared to the interfacial electron-transfer kinetics in all tested SyN samples, irrespective of the ^Tet^Fe content, such that their redox behavior over the time scale of our measurements is controlled primarily by the interfacial electron-transfer process.

### Interpreting the Fitted Redox Parameters in Relation to Residual ^Tet^Fe

To assess whether the observed redox trends were related to structural differences among the samples in terms of residual ^Tet^Fe content, we performed a principal component analysis (PCA). PCA allowed us to evaluate covariation among the fitted redox parameters, including thermodynamic (*E*_H_^0^ and *g*) and kinetic parameters (log *k*_0_), and to compare these trends with residual ^Tet^Fe content. The resulting biplot captures 90.7% of the total variance of our system, with PC1 and PC2 accounting for 65.6% and 25.1%, respectively (Figure 3B). This high level of captured variance indicates that the two-dimensional projection retained most of the variability in the fitted redox parameters and provided a useful basis for evaluating their relationship with the residual ^Tet^Fe content.

In general, the clusters representing the SyN samples were spatially separated, with only SyN-4 and SyN-5 exhibiting minor overlap of the 95% confidence level ellipses (Figure 3). This separation suggests that, although the redox-manipulated samples share common trends, each sample occupied a distinct region of PCA space based on the fitted redox parameters. Notably, SyN-1 is separated from the three ^Tet^Fe-bearing samples along PC1, consistent with the distinction in fitted rate constants between the ^Tet^Fe-free and ^Tet^Fe-bearing materials. The biplot arrows indicated that the samples primarily differentiated along two axes. The close alignment of the ^Tet^Fe and log *k*_0_ arrows (*r* = 0.82) is consistent with the higher rate constants observed for the ^Tet^Fe-bearing samples. Note that while *E*_H_^0^, *k*_0_, and *g* are model-derived parameters entered into the PCA, the residual ^Tet^Fe content is determined independently using Mössbauer spectroscopy, and the alignment of these quantities lends support to the hypothesis that the presence of ^Tet^Fe improves interfacial electron-transfer rates. In contrast, the arrow representing *g* pointed in the opposite direction of ^Tet^Fe (*r* = −0.77), suggesting that the nonideality of the Fe_s_^2+^/Fe_Total_–*E*_H_ relationships increased as residual ^Tet^Fe content decreased.

### Comparison of Redox Properties of Synthetic Nontronites to Reference Smectites

To contextualize the redox behavior of the analyzed SyN samples, we compared their fitted redox parameters to the reference smectites studied previously (Figure 4).^29,32^ This comparison included the apparent parameters (panels A and B, Figure 4) of the pristine minerals (i.e., the as-synthesized SyN and native reference clay minerals) alongside the parameters extracted from the oxidation and reduction curves of the redox-manipulated specimen (panels C–G, Figure 4). Such an approach situates the SyN samples within the known redox parameter space of natural-iron-bearing smectites.

**Figure 4:**
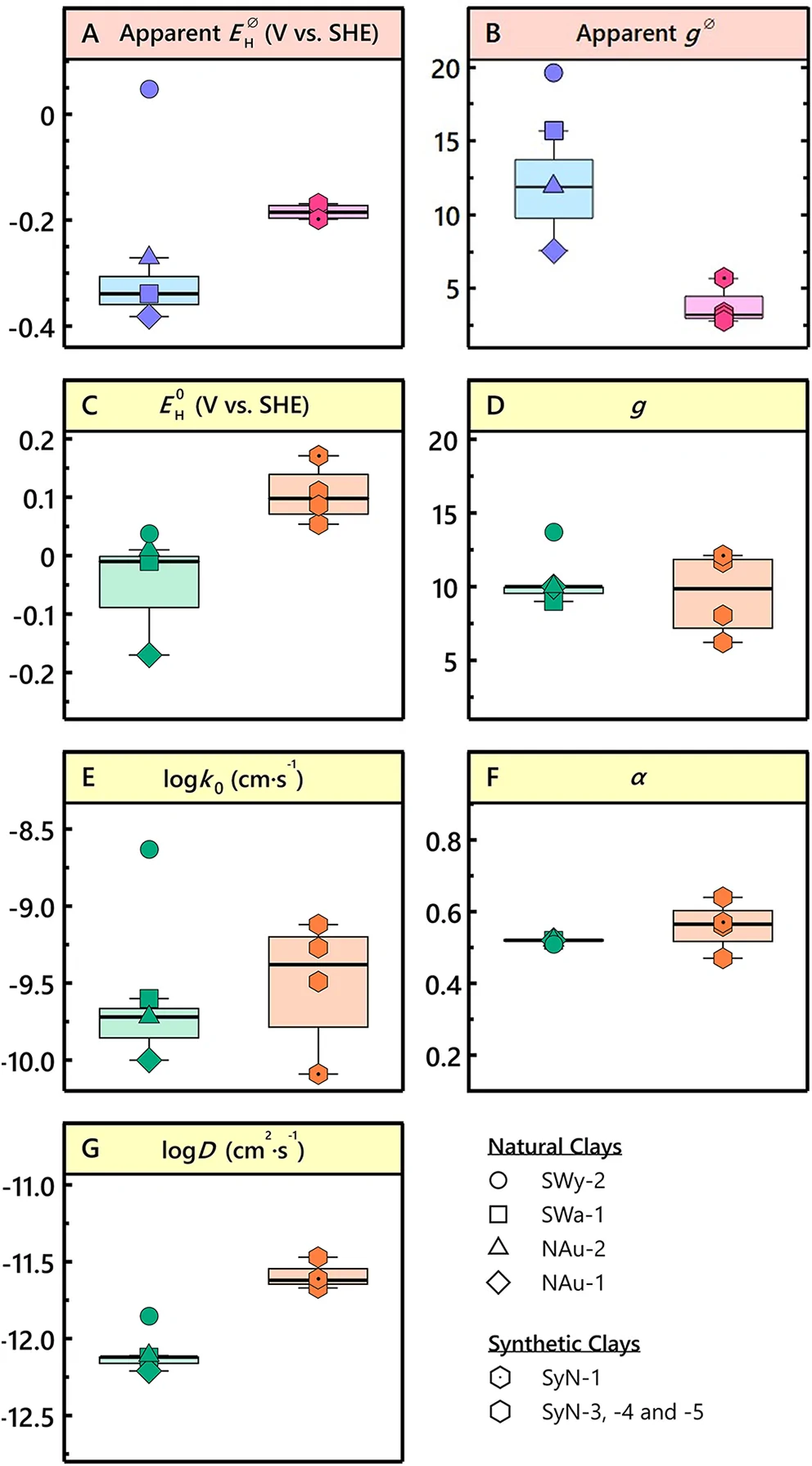
Comparison of fitted redox parameters between natural-iron-rich reference smectites (left part of data panels, blue/green markers) and synthetic nontronites (right-hand side of panels, red/orange markers). Panels A (*E*_H_^Ø^) and B (*g* ^Ø^) show parameters obtained by fitting reduction curves of the pristine clay minerals to eq 2. Panels C (*E*_H_^0^), D (*g*), E (log *k*_0_), F (α), and G (log *D*) show the thermodynamic and kinetic parameters obtained by fitting the process-based model to the reduction and oxidation curves of the redox-manipulated clay minerals. Fitted log *D*-values represent lower bounds (see Table 2). SWy-2 was excluded when generating the box plot for the natural clays, as it chemically differed from the Fe-rich clays.(SWa-1, NAu-1, and NAu-2).

The apparent redox parameters of pristine materials, *E*_H_^Ø^ and *g* ^Ø^, showed a distinction between natural and synthetic smectites. Note that the parameters of SWy-2 (circular marker in Figure 4) are not included in any quantitative comparison here because SWy-2 is chemically distinct from the iron-rich smectites SWa-1, NAu-1, and NAu-2. The *E*_H_^Ø^ values of the reference smectites, SWa-1, NAu-1, and NAu-2 spanned a wider potential range (150 mV, Figure 4A) compared to the as-synthesized SyN samples (30 mV, Figure 4A). The range of *g* ^Ø^ values, describing the interaction between adjacent reactive sites, was also larger and more varied for the reference smectites compared to the SyN samples (Figure 4B). The larger spread in both the *E*_H_^Ø^ and *g* ^Ø^ values in the reference smectites is likely caused by the high degree of chemical heterogeneity compared to synthetic materials.

The comparison of redox parameters for redox-cycled materials is shown in Figure 4C–G. In general, the reference smectites exhibited more negative *E*_H_^0^ values compared to synthetic ones. These *E*_H_^0^-values suggest that the presence of Mg^2+^ and Al^3+^, along with trace amounts of other transition metals within the octahedral sheets of reference smectites, provides additional stability to structural Fe^3+^ compared to the relatively homogeneous octahedral sheets of the synthetic smectites. The interaction parameter, *g*, was largely similar across both sets, though the SyN samples tended toward lower values (Figure 4D). Rate constants for interfacial electron-transfer kinetics, log *k*_0_, were consistently higher for the ^Tet^Fe-bearing SyN samples (SyN-3, -4, and -5) compared to SyN-1 and most reference smectites (Figure 4E), which we speculate is due to the presence of residual Fe in the tetrahedral sheet. The electron transfer coefficients, α, again showed no significant differences between the two groups of smectites (Figure 4F). By contrast, the lower bounds for the apparent diffusion coefficient, *D*, in synthetic smectites exceeded those of the reference smectites (Figure 4G). This difference in the lower bounds of *D* between the SyN samples and the reference smectites likely reflects a more continuous Fe–O–Fe network with fewer structural defects and vacancies, providing a more efficient path for electron hopping than the octahedral sheets, where redox-inactive Al^3+^ and Mg^2+^ substitutions can potentially disrupt charge transfer.^48,49^ Alternatively, the difference could also reflect the relative ease with which the ^Oct^Fe could transition between di- and tri-octahedral arrangements in the absence of octahedral Al^3+^ and Mg^2+^.^21^ Collectively, these comparisons reveal that despite the chemical differences between the reference and synthetic smectites, their redox parameters converge within a narrow range. This convergence of redox properties suggests that the Fe–O–Fe network of the octahedral sheet is the dominant determinant of redox behavior in iron-rich dioctahedral smectites. Compositional variations in ^Tet^Fe occupancy or octahedral substitution only have a minor effect on the parameter set that rationalizes kinetic and thermodynamic descriptors of the Fe redox behavior.

## Environmental Significance

The observed similarity of most of the thermodynamic and kinetic redox parameters derived previously for reference smectites and here, and the minor impact of ^Tet^Fe on the redox properties of redox-cycled synthetic minerals, implies that the redox thermodynamics of Fe in smectites in the environment are largely determined by Fe in the octahedral sheets. We posit that lower *E*_H_^0^ values (0 to −0.20 V) for reference smectites compared to those of redox-cycled synthetic nontronites of +0.10 V are due to the compositional diversity in the Fe coordination environment of the naturally occurring minerals. Geochemical models for nutrient and trace element cycling and pollutant transformation could thus take advantage of including redox-active Fe-rich smectites with a predictably uniform range of reduction potentials. There are, however, outliers to this simplified view of Fe-bearing smectites such as SWy-2 (Figure 4A). Even though these minerals have only low Fe contents of a few wt%, they are more abundant and are hence also relevant contributors to the Fe redox chemistry in the subsurface.

Like previous work involving MEA of reference smectites, their synthetic counterparts studied here confirmed the different redox properties of native or as-synthesized minerals vs those undergoing redox cycling. Even decades after the initial investigations of the Fe redox reactions in clay minerals,^50,51^ it is still unclear whether this observation is the outcome of irreversible structural alterations associated with the first Fe reduction step or a consequence of reductions with dithionite.^52^ A promising approach to resolving this conundrum is to bypass the reduction step entirely by directly synthesizing smectites with a defined Fe^2+^ content, such that the native state of the mineral is already partially or fully reduced.^53,54^ This approach decouples structural changes associated with reduction from those inherent to the mineral’s as-synthesized state, enabling a cleaner interrogation of how the Fe oxidation state and octahedral sheet composition govern redox properties. Applying mediated electrochemical analysis to such synthetic, compositionally tunable smectites offers a promising new avenue to resolve longstanding questions about Fe redox behavior in clay minerals and to establish predictive frameworks for their role in subsurface biogeochemistry.

## Supplementary Material



## Data Availability

Data and code associated with the manuscript are available under 10.25678/000GXQ.

## Notes

Structural formulae and Fe content and elemental compositions of synthetic nontronites, schematic of redox manipulations, methods for mediated electrochemical analyses, sensitivity analysis, and goodness-of-fit information (PDF)
