# Supplementary material for: Redox Properties of Structural Fe in Clay Minerals: 5. The Role of Fe in the Tetrahedral Sheets of Synthetic Smectites
Source: Environ Sci Technol. 2026 Oct 1;60(40):28720–9. doi: 10.1021/acs.est.6c07316 (PMC13674067; doi:10.1021/acs.est.6c07316)
Supplement: Supplementary file 1 [file es6c07316_si_001.pdf]

Supporting Information

Redox Properties of Structural Fe in Clay Minerals: 5. The Role of Fe in the Tetrahedral Sheet of Synthetic Smectites

Vineeth Pothanamkandathil,† Fabien Baron,‡ Anke Neumann,¶,§ Jagannath Biswakarma,‖ Michael Sander,⊥ Thomas B. Hofstetter,*,‖,⊥ and Meret Aeppli*,†

†*School of Architecture, Civil and Environmental Engineering (ENAC), École Polytechnique Fédérale de Lausanne (EPFL), 1015 Lausanne, Switzerland*

‡*Université de Poitiers, CNRS, IC2MP, Poitiers, France*

¶*PSI Center for Nuclear Engineering and Sciences, 5232 Villigen PSI, Switzerland*

§*School of Engineering, Newcastle University, Newcastle upon Tyne, NE1 7RU, United Kingdom*

‖*Eawag, Swiss Federal Institute of Aquatic Science and Technology, 8600 Dübendorf, Switzerland*

⊥*Institute of Biogeochemistry and Pollutant Dynamics (IBP), ETH Zürich, 8092 Zürich, Switzerland*

E-mail: thomas.hofstetter@eawag.ch; meret.aeppli@epfl.ch

10 Pages, 3 Tables

Contents

S1 Summary of the structural composition of the as-synthesized nontronite samples **3**

S2 Schematic of Redox Manipulations **4**

S3 Mediated Electrochemical Analyses **5**

S4 Initial Fe_s^{2+}/Fe_{Total} ratio calculation for simulating oxidation curves **6**

S5 Sensitivity analysis and goodness-of-fit information **7**
Sensitivity analysis 7
Goodness-of-fit 8

References **10**

S1 Summary of the structural composition of the as-synthesized nontronite samples

Table S1 Structural formulae and Fe content of synthetic nontronite samples.

Sample	Structural Formula[a]	^{Oct}Fe (wt%)	^{Tet}Fe (wt%)	Total Fe (wt%)
SyN-1	$Na_{0.38}[Fe^{+3}_{1.91}Al_{0.09}][Si_{3.62}Al_{0.38}]O_{10}(OH)_2$	25	0	25
SyN-2	$Na_{0.38}[Fe^{+3}_{2}][Si_{3.62}Fe^{+3}_{0.38}]O_{10}(OH)_2$	25	5	30
SyN-3	$Na_{0.60}[Fe^{+3}_{2}][Si_{3.40}Fe^{+3}_{0.60}]O_{10}(OH)_2$	25	8	33
SyN-4	$Na_{1.04}[Fe^{+3}_{2}][Si_{2.96}Fe^{+3}_{1.04}]O_{10}(OH)_2$	24	12	36
SyN-5	$Na_{1.09}[Fe^{+3}_{2}][Si_{2.91}Fe^{+3}_{1.09}]O_{10}(OH)_2$	24	13	37

[a]Structural formula determined based on XRD analysis and expressed on a half-unit cell basis.

Table S2 ICP-OES elemental composition of synthetic nontronite samples. All samples were prepared at a clay concentration of 20 g/L.

Sample	ICP-OES				Fe content (at.%)
	Al (mol/L)	Fe (mol/L)	Na (mol/L)	Si (mol/L)	
SyN-1	8.42×10^{-4}	3.40×10^{-3}	3.19×10^{-3}	7.34×10^{-3}	–
SyN-2	–	5.17×10^{-3}	4.52×10^{-3}	1.16×10^{-2}	11.8
SyN-3	–	6.85×10^{-3}	6.28×10^{-3}	1.37×10^{-2}	12.6
SyN-4	–	6.94×10^{-3}	3.93×10^{-3}	8.37×10^{-3}	13.5
SyN-5	–	8.00×10^{-3}	3.90×10^{-3}	1.17×10^{-2}	14.3

S2 Schematic of Redox Manipulations

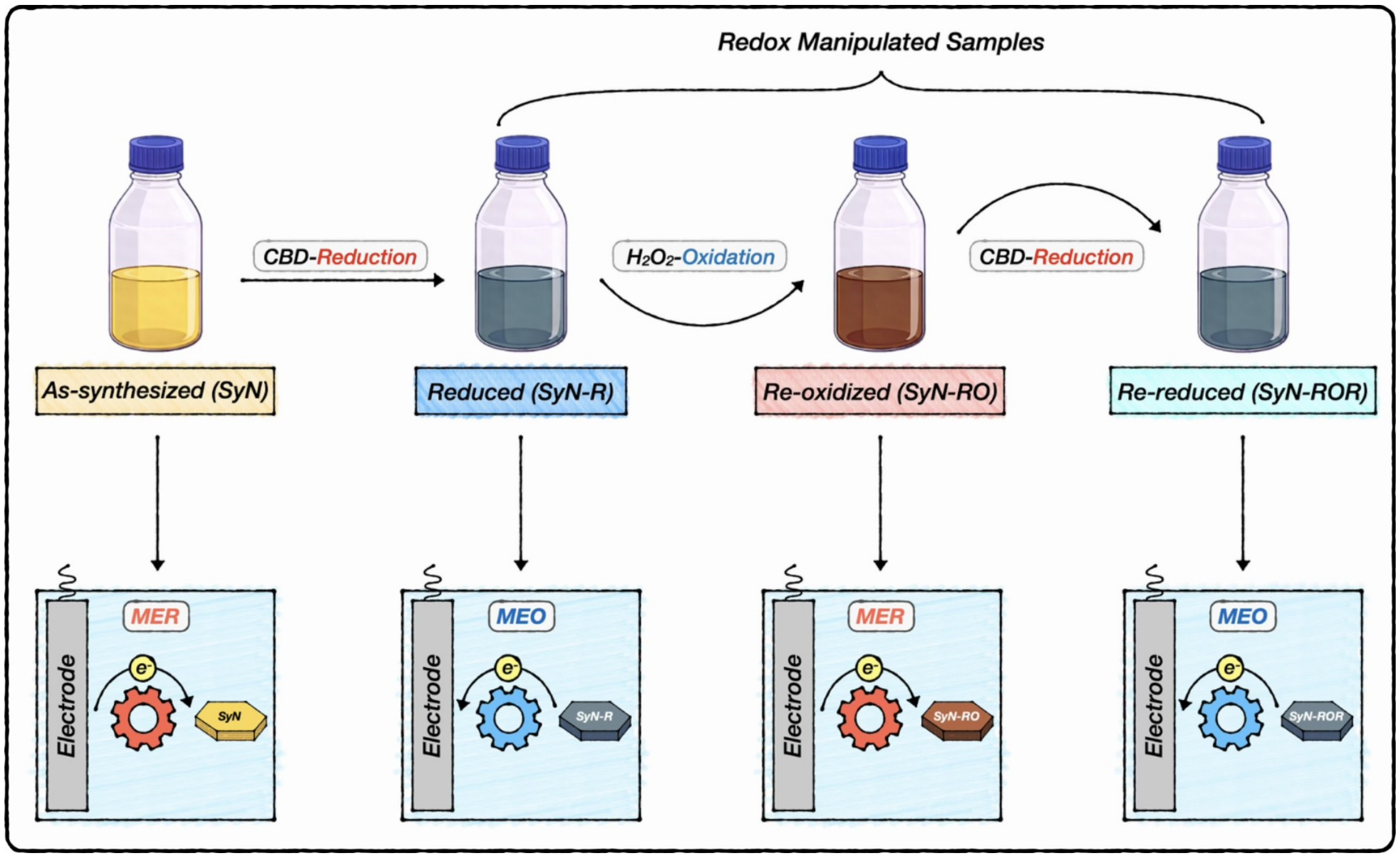

Figure S1 Schematic representation of the redox manipulations applied to the SyN samples and the corresponding mediated electrochemical measurements. Chemical reduction of structural Fe(III) was carried out using the citrate-bicarbonate dithionite (CBD) method, and re-oxidation of structural Fe(II) was carried out using H_2O_2. Images of sample bottles were created using BioRender.

S3 Mediated Electrochemical Analyses

MER and MEO experiments were performed using two eight-channel potentiostats (models 1000B and 1000C, CH Instruments) operated in chronoamperometric mode as described earlier.[1,2] Each electrochemical cell consisted of a 9 mL glassy carbon working electrode (WE) cylinder (GAZ 1, HTW, Germany), a platinum wire counter electrode separated from the WE compartment by a porous glass frit (PORE E tubes; ACE Glass), and an Ag/AgCl reference electrode (Re1B, ALS, Japan). All potentials are reported versus the standard hydrogen electrode (SHE).

Nine electron transfer mediators were selected to cover the applied potential range from −0.57 V to +0.70 V vs. SHE:[3] ethyl viologen (1,1′-bis(ethyl)-4,4′-bipyridyl, $E^0_\mathrm{H} = -0.45$ V), diquat (1,1′-ethylene-2,2′-bipyridyl, $E^0_\mathrm{H} = -0.35$ V), flavin mononucleotide ($E^0_\mathrm{H} = -0.21$ V), cyanomethylviologen (1,1′-bis(cyanomethyl)-4,4′-bipyridyl, $E^0_\mathrm{H} = -0.14$ V), resorufin ($E^0_\mathrm{H} = -0.06$ V), 1,4-naphthoquinone ($E^0_\mathrm{H} = +0.06$ V), DCPIP (2,6-dichlorophenolindophenol, $E^0_\mathrm{H} = +0.22$ V), ferri/ferrocyanide ($Fe^{2+/3+}(CN)_6$, $E^0_\mathrm{H} = +0.43$ V), and ABTS (2,2′-azino-bis(3-ethylbenzothiazoline-6-sulfonic acid), $E^0_\mathrm{H} = +0.70$ V). One- and two-electron transfer mediators were used within ±0.12 V and ±0.06 V of their E^0_H values, respectively. Measurements were performed in pH 7.50-buffered solution, except for experiments with ABTS, which were conducted at pH 6.50 due to its instability at higher pH. Clay suspensions were added from 1 g L^{-1} stock suspensions to the electrochemical cells after equilibration with the respective mediator at each applied potential, with total Fe maintained at 0.1 μmol per cell.

The number of electrons transferred to structural iron ($\mathrm{n}_{\mathrm{Fe}^{\mathrm{III}}_{\mathrm{MER}}}$) was determined from the positive current response according to eq. S1, and the number of electrons transferred from structural iron ($\mathrm{n}_{\mathrm{Fe}^{\mathrm{II}}_{\mathrm{MEO}}}$) from the negative current response according to eq. S2:

$$\mathrm{n}_{\mathrm{Fe}^{\mathrm{III}}_{\mathrm{MER}}} = \frac{1}{F} \int_{t_0}^{t_{end}} I_{red}\, dt \tag{S1}$$

$$\mathrm{n}_{\mathrm{Fe}^{\mathrm{II}}_{\mathrm{MEO}}} = \frac{1}{F} \int_{t_0}^{t_{end}} -I_{ox}\, dt \tag{S2}$$

where I_red and I_ox are the baseline-corrected reductive and oxidative currents (A), respectively, F is the Faraday constant, and t_0 and t_end (s) denote the initial and final integration boundaries for each current peak. To express the electrochemical data as redox profiles, we normalized $\mathrm{n}_{\mathrm{Fe}^{\mathrm{III}}_{\mathrm{MER}}}$ and $\mathrm{n}_{\mathrm{Fe}^{\mathrm{II}}_{\mathrm{MEO}}}$ to $\mathrm{Fe}_\mathrm{Total}$, which was determined electrochemically as the sum of the electron accepting and donating capacities of the sample (eq. S3):

$$\mathrm{Fe}_\mathrm{Total} = \mathrm{EAC} + \mathrm{EDC} \tag{S3}$$

Here, EAC is the electron accepting capacity of the total Fe(III) pool, quantified at −0.570 V vs. SHE and pH 7.50 – conditions sufficient to fully reduce structural Fe(III) in clay minerals – and EDC is the electron donating capacity of the total Fe(II) pool, quantified at +0.700 V vs. SHE and pH 6.50 – conditions sufficient to fully oxidize structural Fe(II) in clay minerals.

S4 Initial Fe_s^{2+}/Fe_{Total} ratio calculation for simulating oxidation curves

The initial Fe_s^{2+}/Fe_{Total} ratio is determined electrochemically using the following equation:

$$\text{Initial}\frac{Fe_s^{2+}}{Fe_{Total}} = \frac{EAC}{EEC} \tag{S4}$$

where EAC (moles) represents the electron accepting capacity and is defined as the number of moles of electrons passed to Fe_s^{3+} during an MER experiment ($n_{Fe^{III}_{MER}}$; **eq. 1**) conducted at –0.570 V vs. SHE and electron exchange capacity (EEC) (moles) represents the electron exchange capacity of the clay mineral:[2]

$$EEC = EAC + EDC \tag{S5}$$

where EDC (moles) represents the electron donating capacity and is defined as the number of moles of electrons extracted from Fe_s^{2+} during an MEO experiment ($n_{Fe^{II}_{MEO}}$; **eq. 2**) conducted at +0.700 V vs. SHE.

S5 Sensitivity analysis and goodness-of-fit information

Sensitivity analysis

The model used to describe the Fe_s^{2+}/Fe_{Total} — E_H relationships contains five fitted parameters (E_H^0, k_0, D, α, and g). To assess how strongly each parameter is constrained by the experimental data, we performed a sensitivity analysis in which each parameter was varied systematically across its fitting range while the remaining four were held at their best-fit values. The sum of squared differences between the simulated and measured redox curves was evaluated at each point (Figure S2).

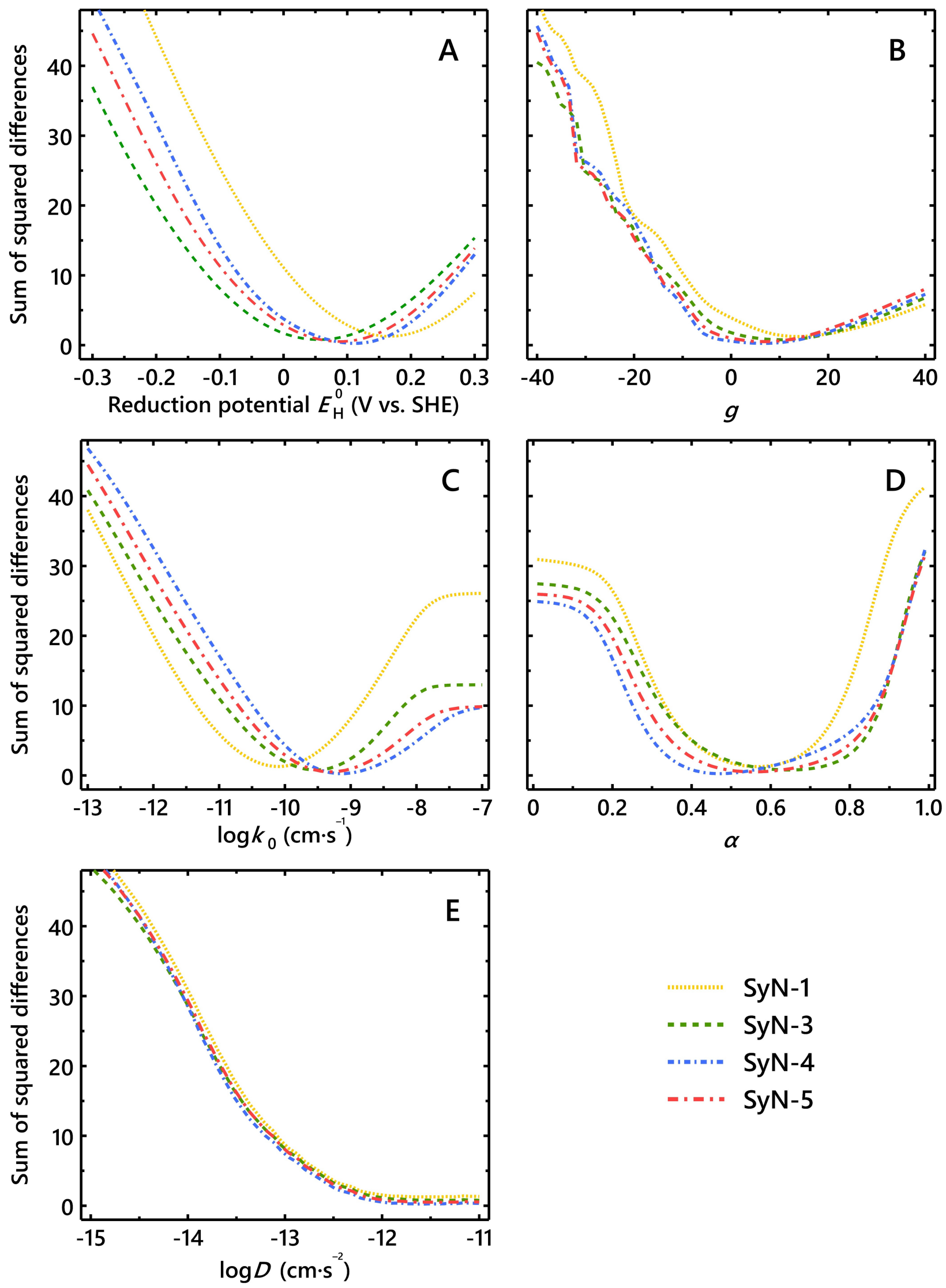

Figure S2 Sensitivity of the sum of squared differences between simulated and experimental redox curves to variation in each fitted parameter: (A) E_H^0, (B) g, (C) log k_0, (D) α, and (E) log D. Each parameter was varied around its fitted optimum while the remaining parameters were held at their best-fit values.

Goodness-of-fit

In addition to the sensitivity analysis, residual plots of the difference between the simulated and measured Fe_s^{2+}/Fe_{Total} values were generated for each redox manipulated SyN sample to assess the quality of the fits beyond the reported r^2 values (Figure S3). The residuals are distributed around zero without systematic trends across the applied potential range, indicating that the model reproduces the shape of the measured redox curves without directional bias. The root mean square errors are provided in Table S3.

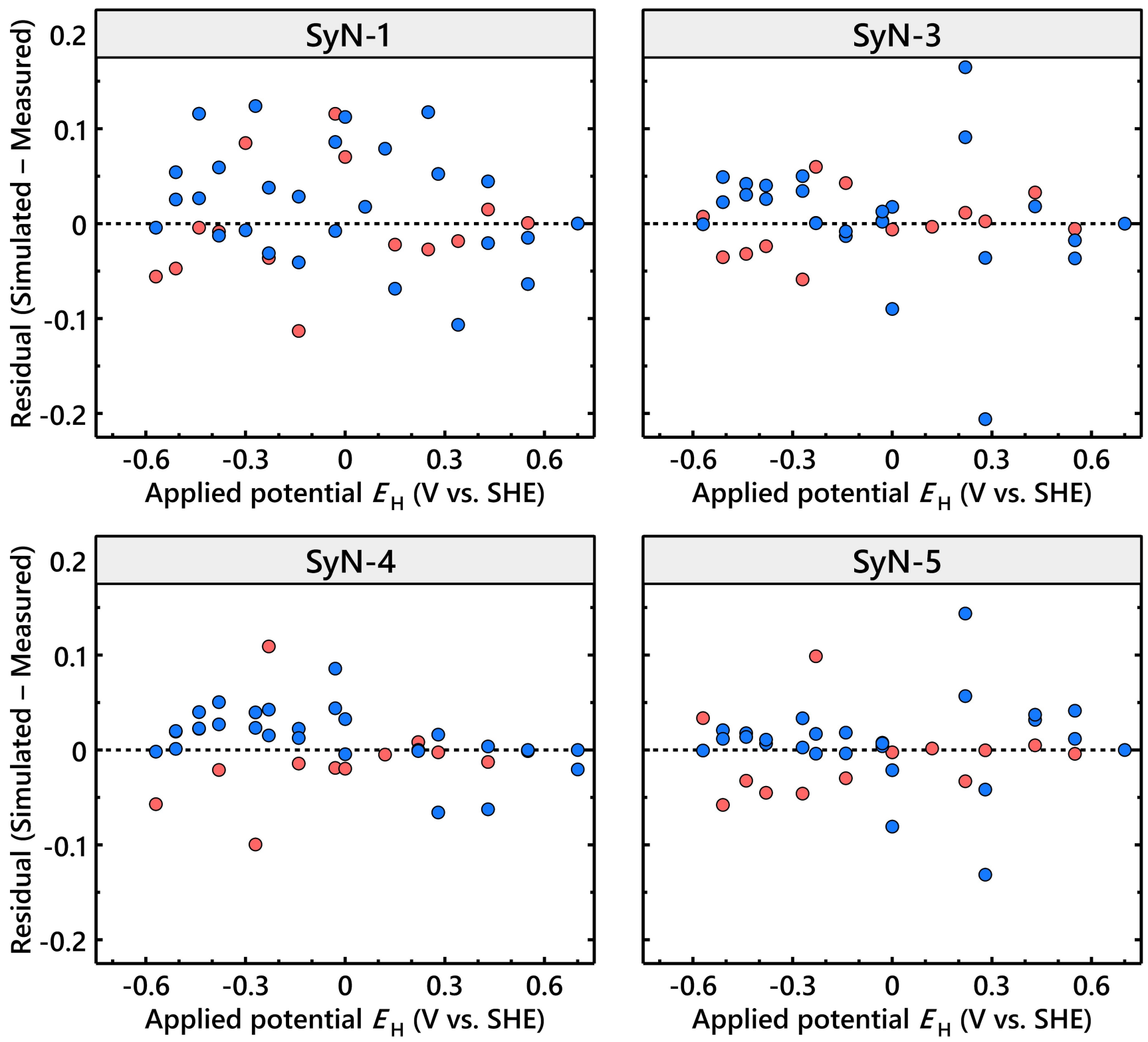

Figure S3 Residuals between simulated and measured Fe_s^{2+}/Fe_{Total} values as a function of applied potential for the redox-manipulated SyN samples. Red symbols denote reduction curves and blue symbols oxidation curves.

Table S3 Root mean square errors (RMSE) between simulated and measured Fe_s^{2+}/Fe_{Total} values for the redox-manipulated SyN samples.

Sample	RMSE		
	**Reduction**	**Oxidation**	**Overall**
SyN-1	0.056	0.061	0.059
SyN-3	0.029	0.061	0.052
SyN-4	0.043	0.033	0.037
SyN-5	0.038	0.045	0.043

References

(1) Sander, M.; Hofstetter, T. B.; Gorski, C. A. Electrochemical analyses of redox-active iron minerals: A review of nonmediated and mediated approaches. *Environ. Sci. Technol.* **2015**, *49*, 5862–5878, https://doi.org/10.1021/acs.est.5b00006.

(2) Gorski, C. A.; Aeschbacher, M.; Soltermann, D.; Voegelin, A.; Baeyens, B.; Marques Fernandes, M.; Hofstetter, T. B.; Sander, M. Redox Properties of Structural Fe in Clay Minerals. 1. Electrochemical Quantification of Electron-Donating and -Accepting Capacities of Smectites. *Environ. Sci. Technol.* **2012**, *46*, 9360–9368, https://doi.org/10.1021/es3020138.

(3) Gorski, C. A.; Klüpfel, L. E.; Voegelin, A.; Sander, M.; Hofstetter, T. B. Redox Properties of Structural Fe in Clay Minerals. 3. Relationships between Smectite Redox and Structural Properties. *Environ. Sci. Technol.* **2013**, *47*, 13477–13485, https://doi.org/10.1021/es403824x.
